# Comparative Study of Two Chondroitin Sulfate/Dermatan Sulfate 4-*O*-Sulfatases With High Identity

**DOI:** 10.3389/fmicb.2019.01309

**Published:** 2019-06-12

**Authors:** Shumin Wang, Tiantian Su, Qingdong Zhang, Jingwen Guan, Jing He, Lichuan Gu, Fuchuan Li

**Affiliations:** National Glycoengineering Research Center and Shandong Provincial Key Laboratory of Carbohydrate Chemistry and Glycobiology, and State Key Laboratory of Microbial Technology, Shandong University, Qingdao, China

**Keywords:** glycosaminoglycan, chondroitin sulfate, dermatan sulfate, sulfated polysaccharide, sulfatase

## Abstract

Chondroitin sulfate/dermatan sulfate (CS/DS) sulfatases are potential tools for structural and functional studies of CD/DS chains. In our previous study, a CS/DS 4-*O*-endosulfatase (endoVB4SF) was identified from a marine bacterium (Wang et al., 2015). Herein, another CS/DS 4-*O*-sulfatase (exoPB4SF) was identified from a *Photobacterium* sp. ExoPB4SF shares an 83% identity with endoVB4SF but showed strict exolytic activity. Comparative studies were performed for both enzymes on the basis of biochemical features, substrate-degrading patterns and three-dimensional structures. exoPB4SF exhibited a wider temperature and pH adaptability and better thermostability than endoVB4SF. Furthermore, exoPB4SF is a strict exolytic sulfatase that only releases the sulfate group from the GalNAc residue located at the reducing end, whereas endoVB4SF preferentially removed sulfate esters from the reducing end toward the non-reducing end though its directional degradation property was not strict. In addition, the structure of endoVB4SF was determined by X-ray crystallography at 1.95 Å. It adopts a globular conformation with two monomers per asymmetric unit. The exoPB4SF structure was constructed by homology modeling. Molecular docking results showed that although the residues around the catalytic center are conserved, the residues at the active site of endoVB4SF adopted a more favorable conformation for the binding of long CS/DS chains than those of exoPB4SF, which may explain why the two highly homogenous sulfatases possessed different action patterns. The results of this study provide insight into the structure-function relationship of CS/DS endo- and exosulfatases for the first time.

## Introduction

Chondroitin sulfate (CS) and dermatan sulfate (DS) are glycosaminoglycan (GAG) sidechains of proteoglycans that are widely present in the extracellular matrix and on cell surfaces (Iozzo, 1998). The backbone of CS chains is composed of -[GlcUA-β1-3-GalNAc]- disaccharide units linked by β1-4-glycosidic bonds, while in DS, the β-D-glucuronic acid (GlcUA) residues are transformed to α-L-iduronic acid (IdoUA) residues through the action of glucuronyl C5 epimerase. Thus, CS and DS usually exist in a hybrid form of CS-DS (Sugahara et al., 2003). The backbone of CS/DS can be modified by various sulfotransferases to produce many different disaccharide structures with unique sulfation patterns, such as the following common CS disaccharide units: O unit (GlcUAβ1-3GalNAc), C unit [GlcUAβ1-3GalNAc(6S)], A unit [GlcUAβ1-3GalNAc(4S)], D unit [GlcUA(2S)β1-3GalNAc(6S)], E unit [GlcUAβ1-3GalNAc(4S, 6S)], and T unit [GlcUA(2S)β1-3GalNAc(4S, 6S)]; and the following DS disaccharide units: iO unit (IdoUAα1-3GalNAc), iA unit [IdoUAα1-3GalNAc(4S)] and iB unit [IdoUA(2S)α1-3GalNAc(4S)] (Silbert and Sugumaran, 2002). These modifications can result in relatively complex CS/DS structures, endowing them with a variety of biological functions (Linhardt and Hileman, 1995). CS/DS chains are structural materials in connective tissues and are key functional molecules regulating various physiological and pathological processes (Linhardt and Toida, 2004), such as cell division, central nervous system development, viral and bacterial infections, and tumor progression (Hardingham and Fosang, 1992; Faissner et al., 1994; Wang et al., 2016). Many studies have shown that CS/DS chains exert these functions through interactions with various proteins, such as adhesion molecules, growth factors and cytokines, where the sulfation pattern of CS/DS chains is crucial for these interactions (Malavaki et al., 2008; Mizumoto et al., 2015; Djerbal et al., 2017).

CS/DS sulfatases specifically catalyze the hydrolysis of sulfate groups from CS/DS chains and are potential tools for structural and functional studies of CS/DS (Sugahara and Kojima, 1996; Ulmer et al., 2014; Wang et al., 2015). These enzymes belong to the conservative arylsulfatase family, whose members carry a C_α_-formylglycine (FGly) residue generated by the oxidation of a conserved cysteine/serine residue at the active site of the enzyme and are essential for the degradation of CS/DS in various organisms, from bacteria to animals (Knaust et al., 1998; Boltes et al., 2001; Berteau et al., 2006). In humans, deficiencies in these enzymes results in a series of physical disorders, such as multiple sulfatase deficiency (MSD) (Czyzewska and Dzialoszynski, 1978; Dierks et al., 2003). Recently, a CS/DS sulfatase was shown to participate in the axon regeneration process of central nervous system injures in mammals through the modification of CS/DS chains (Pearson et al., 2018). However, despite being an important class of enzymes, only a few CS/DS sulfatases from prokaryotes and eukaryotes have been studied in detail, including *N*-acetylgalactosamine-4-*O*-sulfatase (Wang et al., 2015), *N*-acetylgalactosamine-6-*O*-sulfatase and Δ^4,5^hexuronate-2-*O*-sulfatase (Sugahara and Kojima, 1996; Myette et al., 2003; Ulmer et al., 2014).

Most of the identified CS/DS sulfatases are exo-type enzymes, which hydrolyze sulfate esters from the ends of CS/DS poly-/oligosaccharides. The catalytic mechanisms of these enzymes have been well elucidated through structural and biochemical studies of several enzymes, including three human CS/DS exosulfatases: ARSB (4-*O*-sulfatase) (Bond et al., 1997), GALNS (6-*O*-sulfatase) (Rivera-Colon et al., 2012) and IDS (iduronate-2-*O*-sulfatase) (Demydchuk et al., 2017); and two bacterial Δ^4,5^hexuronate-2-*O*-sulfatases from *Pedobacter heparinus* (Raman et al., 2003) and *Bacteroides thetaiotaomicron* (Ulmer et al., 2014). In contrast, endo-type CS/DS sulfatases, which can effectively remove sulfate groups within the CS/DS chains, are rare, with only two endo-4-*O*-sulfatases having been identified from *Bacteroides thetaiotaomicron* (Ulmer et al., 2014) and *Vibrio* sp. (Wang et al., 2015). Compared to exosulfatases, CS/DS endosulfatases are more potent tools for structural and functional studies of CS/DS. However, the detailed action pattern and catalytic mechanism of these enzymes remain to be investigated using biochemical and crystallographic methods.

In this study, a novel exo-type CS/DS 4-*O*-sulfatase (exoPB4SF) was identified from a newly isolated bacterium, *Photobacterium* sp. FC615. Interestingly, this enzyme shares high sequence identity (83%) with the endo-type 4-*O*-sulfatase (endoVB4SF) from *Vibrio* sp. (Wang et al., 2015), providing an ideal model for investigating the different mechanisms of action between exo- and endosulfatases. Herein, a comparative study was performed on the basic biochemical features, substrate-degrading patterns and catalytic mechanisms between the two 4-*O*-sulfatases exoPB4SF and endoVB4SF. The results of this study showed that the CS/DS endosulfatase endoVB4SF has a directional tendency during hydrolysis of sulfate groups, and the crystal structure of CS/DS endosulfatase was determined for the first time to elucidate its mechanism of action.

**Table 1:** Bacterial strains and plasmids.

Strains and plasmids	Description	Source
*Strains*		
*Photobacterium* sp. FC615	A CS-degrading marine bacterium (patented as CGMCC NO. 16918)	
*E. coli* BL21 (DE3)	*F-, ompT, hsdSB (rB-, mB-), dcm, gal, λ (DE3), pLysS, Cm^r^*	Transgen
*Plasmids*		
pET-30a	Expression vector; kanamycin-resistant	Invitrogen
pET-30a-*exopb4sf*	pET-30a, carrying the recombinant protein of exoPB4SF	
pET-30a-*endovb4sf*	pET-30a, carrying the recombinant protein of endoVB4SF	

## Materials and Methods

### Materials

The strains and plasmids used in this study are listed in Table 1. PrimeSTAR^TM^ HS DNA polymerase, restriction endonucleases and other genetic engineering enzymes were purchased from Takara Inc. (Dalian, China). A genomic DNA extraction kit was purchased from Tiangen Biotech Co., Ltd. (Beijing, China). Competent cells and reagents for site-directed mutagenesis were obtained from TransGen Biotech Co., Ltd. (Beijing, China). Reagents for protein crystallization were purchased from Hampton Research (Aliso Viejo, United States). Standard GAG unsaturated disaccharides were purchased from Iduron (Manchester, United Kingdom). DS from porcine skin were purchased from Seikagaku Corp. (Tokyo, Japan). CS-A from bovine cartilage, chondroitinase ABC (CSase ABC) (EC 4.2.2.4), 2-aminobenzamide (2-AB), sodium borohydride and sodium cyanoborohydride (NaBH_3_CN) were purchased from Sigma-Aldrich Inc. The hexasaccharide ΔA-A-A [Δ^4,5^HexUAβ1-3GalNAc(4S)β1-4Gl cUAβ1-3GalNAc(4S)β1-4GlcUAβ1-3GalNAc(4S)] was prepared from CS-A as described below.

### Preparation of the Hexasaccharide ΔA-A-A

An aliquot of commercial CS-A (10 mg) was partially digested with 10 U of highly purified rHCLase (Han et al., 2014) in 50 mM NaH_2_PO_4_-Na_2_HPO_4_ buffer (pH 8.0) in a total volume of 4 ml at 37°C for 10 min. The reaction sample was heated in boiling water for 10 min and then cooled in ice-cold water for 10 min. The digest was fractionated on a Superdex^TM^ Peptide 10/300 GL column (GE Healthcare) with 0.2 mM NH_4_HCO_3_ as the eluent. The hexasaccharide fraction was pooled and further separated on a YMC-Pack PA-G column (YMC-Pack PA, Kyoto, Japan), eluted with a linear gradient from 16 to 460 mM NaH_2_PO_4_ over 60 min at a flow rate of 1.0 ml/min at room temperature. The subfractions were pooled and desalted using a Superdex^TM^ Peptide10/300 GL column followed by repeated lyophilization. The disaccharide compositions of these hexasaccharide subfractions were analyzed by digestion with the CSase ABC followed by HPLC separation on a YMC-Pack PA-G column as described above. The hexasaccharide fraction that yielded a single disaccharide Δ^4,5^HexUAβ1-3GalNAc(4S) was the ΔA-A-A hexasaccharide.

### Sequence Analysis of the Genes and Proteins of exoPB4SF and endoVB4SF

The promoter motifs of the 5′-flanking DNA region upstream to the open reading frames (ORFs) were identified using Primer Premier version 5.0 (PREMIER Biosoft International, Palo Alto, CA, United States) and the Promoter 2.0 Prediction Server. The GC content of the ORFs were calculated using Bio-Edit version 7.0.5.3. The molecular masses of the proteins were estimated using the peptide mass tool on the ExPASy server of the Swiss Institute of Bioinformatics. Secretion signal peptides were identified using the SignalP 4.1 server. An online similarity search of the protein sequence was performed using the BLASTp algorithm. Multiple sequence alignments and phylogenetic analysis were performed using MEGA version 7.0. Sequences of endoVB4SF (GenBank^TM^ accession number: AJK90566) from *Vibrio* sp. FC509 (Wang et al., 2015), Δ^4,5^hexuronate-2-*O*-sulfatase (GenBank^TM^ accession number: AXP31968) from *Photobac- terium* sp. FC615, Δ^4,5^hexuronate-2-*O*-sulfatase (GenBank^TM^ accession number: WP_015808635) from *Pedobacter heparinus* (Myette et al., 2003), Δ^4,5^hexuronate-2-*O*-sulfatase (GenBank^TM^ accession number: NP_810509), *N*-acetylgalactosamine-4-*O*-sulfatase (GenBank^TM^ accession number: AAO78455) and *N*-acetylgalactosamine-6-*O*-sulfatase (GenBank^TM^ accession number: NP_812245) from *Bacteroides thetaiotaomicron* (Ulmer et al., 2014) were downloaded from the NCBI protein sequence database.

### Heterologous Expression and Purification of the exoPB4SF and endoVB4SF Proteins

EndoVB4SF (containing amino acids 20–521) was heterologously expressed in *E. coli* strain BL21 (DE3) as described previously (Wang et al., 2015).

To express the sulfatase exoPB4SF in *E. coli*, the full-length gene encoding exoPB4SF was amplified using the primer pair 5′-GAATTCTTGACGGGCAATCAGCCCGCTG-3′ (forward) and 5′-CTCGAGGGTATAGGCATTAAGTACACTGG-3′ (reverse) and high-fidelity PrimeSTAR^TM^ HS DNA polymerase. Next, the PCR product was inserted into the expression vector pET-30a (+) (Invitrogen). The exoPB4SF protein with a His6 tag added at the C-terminus was expressed as previously described for endoVB4SF (Wang et al., 2015). For protein purification, the bacterial cells were harvested by centrifugation at 6,000 × *g* for 15 min, washed twice using ice-cold buffer A (50 mM Tris-HCl and 150 mM NaCl, pH 8.0), resuspended in buffer A, and disrupted by sonication (50 repetitions, 5 s) in an ice-cold environment. After centrifugation at 15,000 × *g*, 4°C for 30 min, the supernatant was loaded onto a nickel affinity column (nickel-Sepharose^TM^ 6 Fast Flow resin, GE Healthcare) and was further purified via ion exchange (Source 15Q HR 16/10, GE Healthcare) and size-exclusion chromatography (Superdex^TM^ 200 10/300 GL, GE Healthcare) in 10 mM Tris-HCl and 100 mM NaCl, pH 8.0. The purity of the enzymes was assessed by SDS-PAGE using 13.2% polyacrylamide gels followed by staining with Coomassie Brilliant Blue. Protein concentrations were determined using the Folin-Lowry method (Bramhall et al., 1969).

### Activity of exoPB4SF and endoVB4SF Toward Unsaturated Disaccharides and Polysaccharides

To determine the substrate specificities of the enzymes, different types of unsaturated disaccharides were dissolved in deionized water to prepare stock solutions (200 pmol/μl), including the ΔC, ΔA, ΔD, ΔE, and ΔT units. Each stock solution (10 μl) was mixed with 30 μl of 150 mM NaH_2_PO_4_-Na_2_HPO_4_ buffer (pH 8.0), 40 μl of deionized water and 10 μl of appropriately diluted enzyme (0.5 μg/μl), and the solutions were incubated at 30°C for 12 h. Each enzymatic reaction included a negative control in which active enzyme was substituted with boiled inactive enzyme. The enzyme-treated disaccharide samples were heated in boiling water for 10 min and then cooled in ice-cold water for 10 min. After centrifugation at 15,000 × *g* for 15 min, the supernatants were collected and labeled with 2-AB in the presence of NaBH_3_CN, as described by Bigge et al. (1995). Free 2-AB was removed by extraction with chloroform. All of the samples were analyzed by anion-exchange HPLC on a YMC-Pack PA-G column eluted with a linear gradient from 16 to 460 mM NaH_2_PO_4_ over 60 min at a flow rate of 1.0 ml/min at room temperature. The eluates were monitored by measuring the absorbance using a fluorescence detector at excitation and emission wavelengths of 330 and 420 nm, respectively. Disaccharides were identified and quantified by comparison with CS-derived authentic unsaturated disaccharides.

To assess the enzymatic activities of endoVB4SF and exoPB4SF toward CS/DS polysaccharides, CS-A and DS polysaccharides were used as substrates for enzyme digestion. CS-A (30 μg) and DS (30 μg) was digested with exoPB4SF (5 μg), respectively, in 50 mM NaH_2_PO_4_-Na_2_HPO_4_ buffer (pH 8.0) in a total volume of 100 μl, at 30°C for over 72 h, with the addition of 5 μg of fresh enzyme every 24 h two times. Subsequently, the reaction samples were heated in boiling water for 10 min and then cooled in ice-cold water for 10 min. Next, the sample was digested with the CSase ABC for disaccharide composition analysis. The digest was analyzed by gel filtration chromatography on a Superdex^TM^ Peptide 10/300 GL column. The mobile phase was 0.20 M NH_4_HCO_3_ at a flow rate of 0.4 ml/min, and the eluted fractions were monitored at 232 nm using a UV detector.

To analyze the enzymatic activity of exoPB4SF toward DS tetrasaccharides, 15 μg of DS tetrasaccharides [Δ^4,5^HexUAα1-3GalNAc(4S)β1-4IdoUAα1-3GalNAc(4S)] were digested with exoPB4SF (5 μg) in 50 mM NaH_2_PO_4_-Na_2_HPO_4_ buffer (pH 8.0) in a total volume of 100 μl at 30°C for over 6 h. Subsequently, the reaction samples were heated in boiling water for 10 min and then cooled in ice-cold water for 10 min. After centrifugation at 15,000 × *g* for 15 min, the supernatants were analyzed by anion-exchange HPLC on a YMC-Pack PA-G column eluted with a linear gradient from 16 to 460 mM NaH_2_PO_4_ over 60 min at a flow rate of 1.0 ml/min at room temperature. The eluates were monitored by measuring the absorbance using an ultraviolet detector at 232 nm.

### Biochemical Characterization of endoVB4SF and exoPB4SF

The biochemical characteristics of endoVB4SF were reported in a previous study (Wang et al., 2015). To determine the optimal temperature of exoPB4SF, an aliquot of the ΔA unit (10 nmol) was incubated with 0.1 μg of exoPB4SF in 50 mM NaH_2_PO_4_-Na_2_HPO_4_ buffer (pH 8.0) in a total volume of 100 μl at temperatures ranging from 0 to 70°C for 10 min. After the optimum temperature was determined, the optimal pH assay was carried out in buffers at different pH values, including 50 mM HAc-NaAc buffer (pH 5.0–6.0), 50 mM NaH_2_PO_4_-Na_2_HPO_4_ buffer (pH 6.0–8.0), and 50 mM Tris-HCl buffer (pH 7.0–10.0) at 30°C for 10 min. The effects of chemical agents, including various metal ions, EDTA and DTT (5 mM) on the reaction rate of exoPB4SF were investigated under the optimal reaction conditions (50 mM NaH_2_PO_4_-Na_2_HPO_4_ buffer pH 8.0 at 30°C). Finally, to investigate the thermostability of exoPB4SF, the enzyme was preincubated in 50 mM NaH_2_PO_4_-Na_2_HPO_4_ buffer (pH 8.0) from 0 to 60°C for 0.5–24 h, after which the residual enzymatic activity was determined under the optimum conditions. All reactions were performed in triplicate, and enzyme-treated samples were analyzed by anion-exchange HPLC on a YMC-Pack PA-G column as described above. The eluates were monitored by measuring the absorbance at 232 nm.

The enzymatic activity of exoPB4SF against the ΔA unit was determined under the optimum conditions. An aliquot of the ΔA unit (30 nmol) was incubated with 1 μg of exoPB4SF in 50 mM NaH_2_PO_4_-Na_2_HPO_4_ buffer (pH 8.0) in a total volume of 500 μl at 30°C. At various time intervals (up to 30 min), 30 μl aliquots were withdrawn in duplicate, boiled for 10 min, and then cooled in ice-cold water for 10 min. After centrifugation at 15,000 × *g* for 15 min, the supernatant fluid of each digest was collected and analyzed using anion-exchange HPLC on a YMC-Pack PA-G column. One unit of enzyme was defined as the amount of enzyme required to produce 1 μmol of free sulfate per minute.

### Determination of the Catalytic Direction of endoVB4SF and exoPB4SF

To determine the substrate-degrading orientation of endoVB4SF, aliquots of the hexasaccharide ΔA-A-A (2 μg) purified from CS-A were treated with 0.1 μg of endoVB4SF in 50 mM NaH_2_PO_4_-Na_2_HPO_4_ buffer (pH 8.0) in a total volume of 240 μl at 30°C. At the indicated time points, an aliquot of 30 μl was withdrawn and immediately heated at 100°C to denature the enzyme. The digests were labeled with 2-AB and analyzed by anion-exchange HPLC on a YMC-Pack PA-G column as described above. To further determine the structures of the digested mixtures, ΔA-A-A (10 μg) was partially digested by endoVB4SF, and the digest was fractionated on a YMC-Pack PA-G column. Two intermediates (S2 and S1), with a final product S0, were individually pooled and desalted for sequencing. The disaccharide compositions of the three products (50 pmol each) were first analyzed through CSase ABC digestion and 2-AB labeling followed by anion-exchange HPLC on a YMC-Pack PA-G column as mentioned above. To analyze the disaccharide located at the non-reducing end of S2 or S1, the sample (50 pmol) was labeled with 2-AB and purified by paper chromatography (Wang et al., 2017), after which the 2-AB-labeled sample was digested with CSase ABC and analyzed by HPLC. To investigate the sequence of the tetrasaccharide at the non-reducing end of the hexasaccharide, the sample was reduced with sodium borohydride and then digested with an exo-type CS/DS lyase HCDLase (Wang et al., 2017) at 30°C in 50 mM NaH_2_PO_4_-Na_2_HPO_4_ buffer (pH 8.0) for 6 h. The digest was labeled with 2-AB and analyzed by HPLC as described above.

To investigate the action pattern of exoPB4SF, a similar time-course assay and product-sequencing analysis was performed for exoPB4SF as described for endoVB4SF.

### Crystallization, Data Collection and Structure Refinement

endoVB4SF was purified and concentrated to ∼10 mg/ml. Crystallization conditions were first screened by sitting drop vapor diffusion using crystallization screening kits (Hampton Research) at 20°C. Crystal optimization was performed using the hanging drop vapor diffusion method (McPherson and Gavira, 2014). The hanging drop was prepared by mixing equal volumes of the protein solution and the reservoir solution containing 100 mM HEPS (pH 7.5) and 20% (w/v) PEG 10000. For data collection, crystals were flash-frozen in liquid nitrogen, with 15–20% (v/v) ethylene glycol used as cryoprotectant. The X-ray diffraction data sets were collected at 100K on the beam line BL17U at the Shanghai Synchrotron Radiation Facility (Shanghai, China) equipped with an ADSC Q315r CCD-detector.

The X-ray diffraction data were integrated and scaled using the HKL-2000 program suite (Otwinowski and Minor, 1997). The endoVB4SF structure was resolved by molecular replacement using Phaser from the CCP4 suit of programs (Winn et al., 2011) using the sulfatase (PDB code: 2QZU) as the search model. Model building and refinement was performed using COOT (Emsley and Cowtan, 2004) and PHENIX (Adams et al., 2002), respectively. The final *R*-values were Rwork = 18.54% and Rfree = 21.40% based on a subset of 5% of the reflections.

**Table 2:** X-ray data collection and refinement statistics.

Data collection	endoVB4SF
Space group	C2
Unit-cell parameters	
a, b, c (Å)	116.426, 107.732, 87.569
α, β, γ (°)	90.000, 104.036, 90.000
Wavelength (Å)	0.9870
Resolution (Å)	50.00-1.95 (2.02-1.95)
Measured reflections	267,459
Unique reflections	75,093
Completeness (%)	99.2 (99.6)
Redundancy	3.6 (3.6)
*I/σ(I)*	26.21 (4.72)
*R_merge_* (%)*^a^*	9.2 (41.1)
Wilson *B*-value (Å^2^)	33.19
Refinement	
*R*_working_ (%)	18.54
*R*_free_ (%)	21.40
Protein molecules in ASU*^b^*	2
Protein residues	949
Metal ion	2
Solvent	525
R.m.s.d. from ideal geometry	
Bond length (Å)	0.008
Bond angles (°)	1.271
Mean *B*-factors (Å^2^)	30.40
Ramachandran plot (%)	
Favored	96.49
Allowed	3.51
Outliers	0.00

*The coordinates and structure factors have been deposited in the Protein Data Bank with the accession code 6J66. ^a^R_merge_ = Σ_hkl_Σ_i_|I_i_(hkl) - <I(hkl)>|/Σ_hkl_Σ_i_I_i_(hkl). Where <I(hkl)> is the mean intensity of multiply recorded reflections. ^b^ASU means asymmetric units.*

The diffraction data collection and refinement statistics are listed in Table 2. The final model was checked and validated using PROCHECK (Laskowski et al., 1996), which indicated that the model was of good quality. Structure graphics were illustrated with the PyMOL (Lill and Danielson, 2011) molecular visualization system. The atomic coordinates and structure factors of endoVB4SF were deposited in the Protein Data Bank with accession code 6J66.

The crystallization conditions of exoPB4SF were screened using the same method for endoVB4SF. The exoPB4SF crystalized in reservoir solution containing 0.19 mM CYMAL-7, 0.1 M HEPES (pH 7.5) and 40% (w/v) PEG 400, but the crystal was snowflake-shaped and small. Although the crystallization conditions were optimized by adjusting pH and concentrations of PEG 400, the quality of crystals was not good enough for structure determination even using fragments of exoPB4SF. Therefore, its three-dimensional structure was constructed through structural modeling using ITASSER (Zhang, 2008; Roy et al., 2010; Yang et al., 2015).

### Biochemical Analysis of the Monomer and Dimer endoVB4SF

To analyze if the N-terminal Cys residue contributed to the dimerization of the sulfatase, the mutants of endoVB4SF-C27A and corresponding exoPB4SF-Q5C were produced using a Fast Mutagenesis System Kit (TransGen) as previously described by Prabhakar et al. (2005) The plasmids were amplified using the primer pairs 5′-ATGGTGACTGCTGGCGCGTCGGCTAC-3′ (forward, endoVB4SF-C27A) and 5′-CGCGCCAGCAGTCACC ATGGTTGC-3′ (reverse, endoVB4SF-C27A), and 5′-TTCTTGA CGGGCAATTGCCCCGCTG-3′ (forward, exoPB4SF-Q5C) and 5′-GCAATTGCCCGTCAAGAATTCGGATCCG-3′ (reverse, exoPB4SF-Q5C). The plasmids of the mutants were amplified by transferring them to DMT Chemically Competent Cells (TransGen), and mutated clones were confirmed by DNA sequencing at Sangon Biotech Co., Ltd. (Shanghai, China). Next, the correct mutants were transferred into *E. coli* BL21 (DE3) cells. endoVB4SF-C27A and exoPB4SF-Q5C were expressed and purified as described above for the wild-type enzyme.

The purified endoVB4SF, exoPB4SF, endoVB4SF-C27A and exoPB4SF-Q5C proteins were detected at 280 nm through size-exclusion chromatography (Superdex^TM^ 200 10/300 GL, GE Healthcare) eluted with buffer (10 mM Tris-HCl and 100 mM NaCl, pH 8.0) with or without 5 mM DTT.

### Enzyme-Substrate Molecular Docking

The structures of endoVB4SF and exoPB4SF were manipulated using the “Protein Preparation Wizard” workflow in Maestro. The primary manipulations involved the removal of all water molecules, protonation, and optimization based on the OPLS_2005 force field (Petrovic et al., 2010). The potential acting site near the metal ion was discovered by the SiteMap module, after which a docking grid was generated using the “Receptor Grid Generation” module in Maestro (Schrödinger, 2015b). The grid encloses a box centered on the binding site with a dimension of 10 × 10 × 10 (x × y × z, Å). A scaling factor of 0.8 was set for van der Waals radii of receptor atoms, with a partial atomic charge of less than 0.15. Standard precision of Glide docking procedure (Glide-SP) was used for docking the compounds to the binding site of protein (Schrödinger, 2015a), and the best docking pose of the compound was retained based upon Glide scoring function (G-score).

## Results

### Comparison of the Gene and Protein Sequences of exoPB4SF and endoVB4SF

The putative CS/DS 4-*O*-exosulfatase gene *exopb4sf* (GenBank^TM^ accession number: MK282892) from *Photobacterium* sp. FC615 is 1470 bp in length and has a GC content (G + C%) of 49%. The *exopb4sf* gene encodes a protein of approximately 56.1 kDa composed of 490 amino acids. The isoelectric point (pI) of *exopb4sf* is 5.2, and the SignalP 4.1 analysis showed that the *exopb4sf* protein does not contain an N-terminal signal peptide. In contrast, the CS/DS 4-*O*-endosulfatase gene *endovb4sf* (GenBank^TM^ accession number AJK90566) from *Vibrio* sp. FC509, which shares high sequence identity (83%) with *exopb4sf* (Supplementary Figure S1), is 1566 bp in length, encoding a 60 kDa protein that contains an N-terminal signal peptide of 19 amino acids (Wang et al., 2015).

**FIGURE 1:**
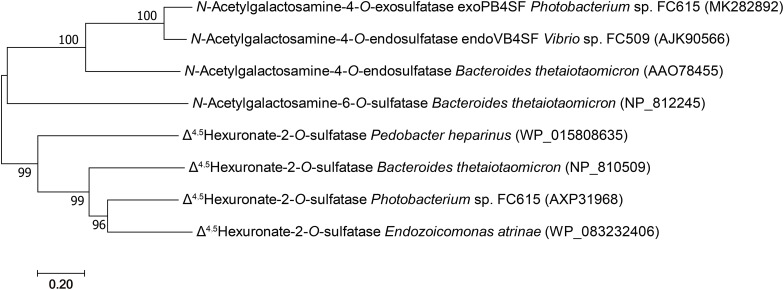
Phylogenetic analysis of the characterized bacterial CS/DS sulfatases. The numbers on the branches indicate the bootstrap confidence values from 1,000 replicates. The bar is equal to the distance corresponding to 1 amino acid substitution per 10 amino acid residues. The neighbor-joining tree was constructed using MEGA.

The results of a neighbor-joining phylogenetic tree analysis showed that exoPB4SF clustered to form a single clade with 4-*O*-sulfatase (Figure 1), resulting in it being preliminarily identified as CS/DS 4-*O*-sulfatase.

### Recombinant Expression of exoPB4SF and endoVB4SF in *E. coli*

The full length sequences of the exoPB4SF and endoVB4SF genes were amplified directly from the genomic DNA of *Photobacterium* sp. FC615 and *Vibrio* sp. FC509, respectively and were recombinantly expressed as described previously (Wang et al., 2015). The results of an SDS-PAGE analysis indicated that BL21 (DE3) cells harboring the pET-30a-*exopb4sf* and pET-30a-*endovb4sf* plasmids yielded ∼4.5 and ∼5 mg/liter of soluble protein with the correct molecular mass, respectively. The crude enzymes were extracted from cultures of the host cells and further purified as described under “*Experimental Procedures.*” Finally, the purity and concentrations of both enzymes was up to 95% and ∼2 mg/ml, respectively (Supplementary Figure S2). The purified enzymes were stored at −80°C for the subsequent biochemical and crystallographic studies.

### Comparison of exoPB4SF and endoVB4SF Activities Toward Unsaturated Disaccharides and Polysaccharides

**FIGURE 2:**
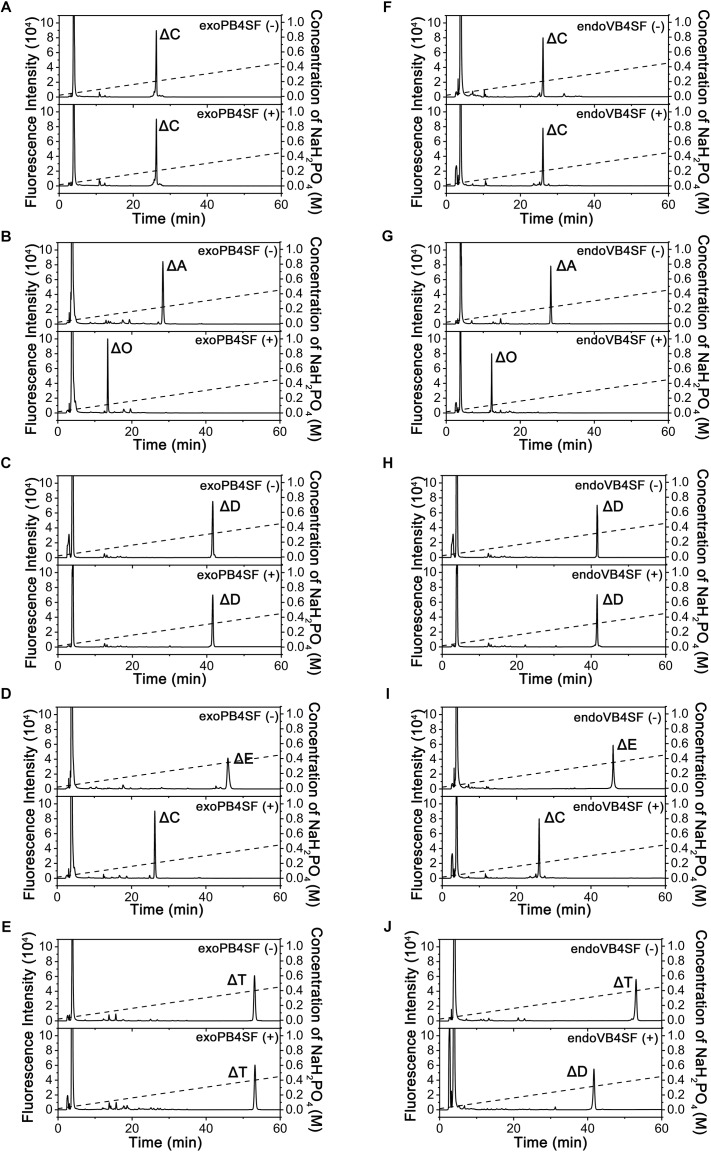
Analysis of the final products from the digestion of CS/DS disaccharides by exoPB4SF and endoVB4SF. Unsaturated CS/DS disaccharides ΔC **(A,F)**, ΔA **(B,G)**, ΔD **(C,H)**, ΔE **(D,I)**, ΔT **(E,J)** were exhaustively digested without (*top*) or with (*bottom*) exoPB4SF **(A–E)** and endoVB4SF **(F–J)**, respectively, labeled with 2-AB, and then analyzed by anion-exchange HPLC as described under “*Experimental Procedures.*” The elution positions of the following standard oligosaccharides are indicated: Δ*O* (Δ^4,5^HexUAβ1-3GalNAc), Δ*C* [Δ^4,5^HexUAβ1-3GalNAc(6S)], Δ*A* [Δ^4,5^HexUAβ1-3GalNAc(4S)], Δ*D* [Δ^4,5^HexUA(2S)β1-3GalNAc(6S)], Δ*E* [Δ^4,5^HexUAβ1-3GalNAc(4S,6S)], and Δ*T* [Δ^4,5^HexUA(2S)β1-3GalNAc(4S,6S)].

To compare the specific activities of exoPB4SF and endoVB4SF toward CS/DS oligo- and polysaccharides, five types of unsaturated CS disaccharides (ΔC, ΔA, ΔD, ΔE and ΔT) with distinct sulfation patterns were individually treated with or without exoPB4SF and endoVB4SF. The results showed that both exoPB4SF and endoVB4SF could effectively remove the 4-*O*-sulfate group from the GalNAc residue of ΔA to yield a non-sulfated ΔO (Figure 2B,G) but no effect was observed toward ΔC with a 6-*O*-sulfate group (Figure 2A,F). Furthermore, for disulfated disaccharides, both enzymes could completely transfer ΔE with 4-*O*- and 6-*O*-sulfate groups at the GalNAc residue to ΔC (Figure 2D,I), although neither enzyme affected ΔD with 2-*O*- and 6-*O*-sulfate groups at the Δ^4,5^hexuronate and GalNAc residues, respectively (Figure 2C,H). In addition, both enzymes did not show any activity toward heparin/heparin sulfate disaccharides (data not shown). Taken together, these results clearly show that exoPB4SF, similar to endoVB4SF, is a CS/DS 4-*O*-sulfatase that specifically hydrolyzes the 4-*O*-sulfate group at GalNAc residues. Notably, unlike endoVB4SF, exoPB4SF could not hydrolyze the 4-*O*-sulfate group of ΔT with three sulfate groups at C4 and C6 of GalNAc and C2 of Δ^4,5^hexuronate (Figure 2E,J), suggesting that the high sulfation of ΔT strongly inhibits the activity of exoPB4SF but not endoVB4SF.

To investigate if exoPB4SF can hydrolyze the 4-*O*-sulfate groups within CS/DS chains, the bovine cartilage-derived polysaccharide CS-A which contains about 67% of A unit and some minor disaccharides C unit and O unit, and DS which contains more than 90% A unit (Wang et al., 2015) were exhaustively treated with this enzyme, and the disaccharide composition of the resulting mixture was analyzed by HPLC as described under “*Experimental Procedures.*” The results showed that the proportion of non-sulfated disaccharide ΔO was not significantly increased by the treatment with exoPB4SF (Supplementary Figure S3A), indicating that the 4-*O*-sulfate groups of GalNAc residues within CS/DS polysaccharide chains cannot be effectively removed by exoPB4SF. Thus, exoPB4SF is a strict exolytic 4-*O*-sulfatase in contrast with the endolytic activity of endoVB4SF (Supplementary Figure S3B) (Wang et al., 2015). However, exoPB4SF could hydrolyze the C-4 sulfate on the reducing end of the DS tetrasaccharides [Δ^4,5^HexUAα1-3GalNAc(4S)β1-4IdoUAα1-3GalNAc(4S)] indicating that exoPB4SF is an exolytic sulfatase that could act on oligosaccharides (Supplementary Figures S3C,D).

### Comparison of the Basic Enzymatic Properties of exoPB4SF and endoVB4SF

**FIGURE 3:**
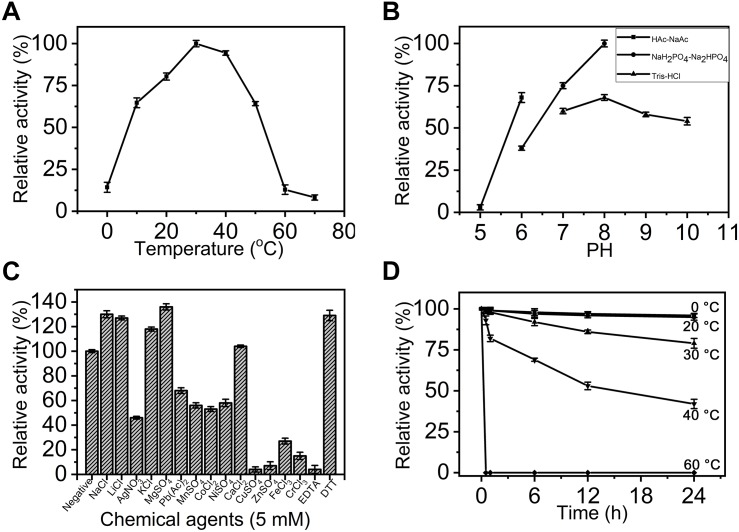
Biochemical characterization of recombinant exoPB4SF. **(A)** Effect of temperature. The enzyme activity of exoPB4SF was determined using the disaccharide ΔA as substrate in 50 mM NaH_2_PO_4_-Na_2_HPO_4_ buffer (pH 8.0) at different temperatures for 10 min. The data are shown as the percentages of the activity obtained at 30°C (100%). **(B)** Effect of pH. The activity of exoPB4SF toward ΔA was measured in buffers with pH values from 5 to 10 at 30°C for 10 min. The data are shown as the percentages of the activity obtained in NaH_2_PO_4_-Na_2_HPO_4_ buffer (pH 8.0) (100%). **(C)** Effects of chemical agents. The activity of exoPB4SF against ΔA was measured in NaH_2_PO_4_-Na_2_HPO_4_ buffer (pH 8.0) containing 5 mM of various chemical agents at 30°C for 10 min. Data are shown as the percentage of the activity of that obtained in the buffer without tested other agents. **(D)** Thermostability of exoPB4SF. The enzyme in 50 mM NaH_2_PO_4_-Na_2_HPO_4_ buffer (pH 8.0) was preincubated for 0–24 h at temperatures from 0 to 60°C, and the residual activity toward ΔA was estimated at 30°C. The data are shown as the activity relative to that of untreated exoPB4SF. Error bars represent means of triplicates ± SD.

To compare the basic enzymatic properties of exoPB4SF and endoVB4SF, the effects of temperature, pH and chemical agents on the activity of exoPB4SF were investigated using ΔA as substrate. Similar to endoVB4SF (Wang et al., 2015), exoPB4SF exhibited the maximal rate at 30°C (Figure 3A), but it possessed a high relative activity (>60%) over a wide range of temperatures, from 10 to 50°C, which was notably different from the high sensitivity of endoVB4SF to different temperatures (Wang et al., 2015). The optimal pH value for exoPB4SF activity was pH 8.0 in 50 mM NaH_2_PO_4_-Na_2_HPO_4_ buffer (Figure 3B), which was the same as that observed for endoVB4SF (Wang et al., 2015). However, it should be noted that the relative activity of exoPB4SF in 50 mM Tris-HCl buffer was low but relatively stable over a pH range of 8.0–10.0 (Figure 3B), whereas that of endoVB4SF quickly decreased as the pH increased from 8.0 to 10.0 in the same buffer (Wang et al., 2015). In addition, the enzymatic activity of exoPB4SF was significantly promoted by some monovalent and divalent cations, including Na^+^, Li^+^, K^+^, Mg^2+^, and Ca^2+^ (Figure 3C), whereas no metal ion notably stimulated the activity of endoVB4SF (Wang et al., 2015). The divalent metal ion-chelating agent EDTA strongly inhibited the activity of exoPB4SF (Figure 3C) but did not significantly affect that of endoVB4SF (Wang et al., 2015). In contrast, the reducing agent DTT significantly promoted the activity of both enzymes (Figure 3C). Thermostability analyses revealed that exoPB4SF retained 80% activity when incubated in 50 mM NaH_2_PO_4_-Na_2_HPO_4_ buffer (pH 8.0) at 30°C for 24 h (Figure 3D), demonstrating that exoPB4SF was more stable than endoVB4SF, the catalytic activity of which decreased dramatically when incubated in the same buffer at 30°C for 4 h.

The enzymatic activity of exoPB4SF was determined under the optimum conditions of 30°C in 50 mM NaH_2_PO_4_-Na_2_HPO_4_ buffer (pH 8.0) using ΔA unit as a substrate. The specific activity of exoPB4SF was 1.37 units/mg of protein, slightly lower than that of endoVB4SF (2.01 units/mg) (one unit of enzyme was defined as the amount of enzyme required to produce 1 μmol of free sulfate per minute) (Table 3). However, the wider temperature and pH adaptability and much better thermostability of exoPB4SF indicated that its structure may be more stable than that of endoVB4SF.

**Table 3:** Purification of recombinant exoPB4SF.

	exoPB4SF		
	Total protein (mg)	Total activity (milliunits)	Specific activity (milliunits/mg)
Crude protein	64.3 ± 4.7	87488	1367 ± 62
Purified protein	12.5 ± 1.2	35712	2857 ± 46

*Results are the means ± SD for at least two experiments.*

### Degradation Pattern Assay of exoPB4SF and endoVB4SF Toward CS-A Hexasaccharide

**FIGURE 4:**
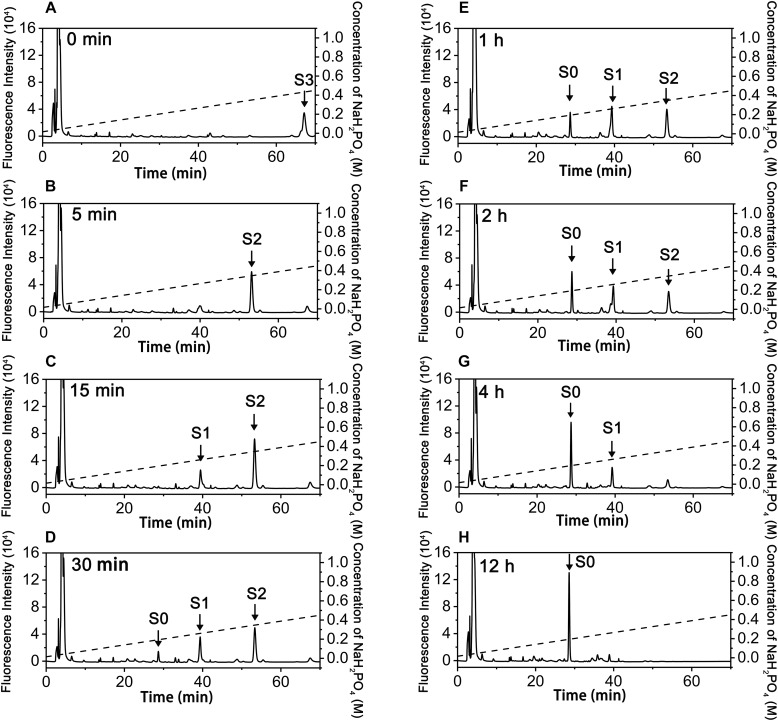
Analysis of products of hexasaccharide ΔA-A-A digested by endoVB4SF. The hexasaccharide ΔA-A-A was digested by endoVB4SF for 0 **(A)**, 5 **(B)**, 15 **(C)**, 30 min **(D)**, 1 **(E)**, 2 **(F)**, 4 **(G)**, or 12 h **(H)**, after which they were labeled with 2-AB and analyzed by anion-exchange HPLC as described under “*Experimental Procedures.*” The elution positions of the resulting hexasaccharide fractions are indicated by *S0*, *S1*, *S2*, and *S3*.

To investigate how exoPB4SF and endoVB4SF act on large substrates with internal 4-*O*-sulfate groups, a hexasaccharide ΔA-A-A [Δ^4,5^HexUAβ1-3GalNAc(4S)β1-4GlcUAα1-3GalNAc (4S)β1-4GlcUAβ1-3GalNAc(4S)] was isolated from CS-A after partial digestion with CS lyase rHCLase (Han et al., 2014) as described under “*Experimental Procedures.*” In a time-course assay, the hexasaccharide ΔA-A-A was treated with exoPB4SF or endoVB4SF for varying times, and the digests were individually analyzed by HPLC on a YMC-Pack PA-G column. As shown in Figure 4, the parental hexasaccharide ΔA-A-A (S3) was quickly transformed to S2 (Figure 4A,B), after which the S2 peak gradually decreased as two earlier-eluting peaks, S1 and S0, correspondingly increased (Figure 4C–F). As the treatment time increased, the intermediates S2 and S1 were finally transferred to the final product S0 (Figure 4G,H). These results suggested that the sulfate groups of the parental hexasaccharide ΔA-A-A were sequentially removed by the action of endoVB4SF. Thus, we presumed that endoVB4SF hydrolyzed the sulfate esters from substrates regularly, not randomly.

**FIGURE 5:**
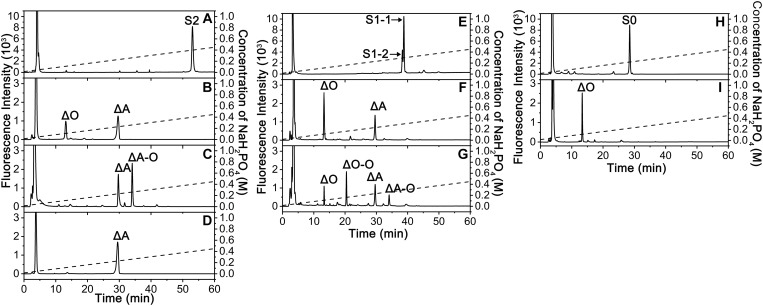
Sequencing analysis of the S2, S1, and S0 products derived from the digestion of ΔA-A-A by endoVB4SF. **(A)** The 2-AB labeled fraction S2; **(B)** The fraction S2 was degraded with the CSase ABC. **(C)** The 2-AB labeled S2 was degraded with the CSase ABC; **(D)** The fraction S2 was reduced with sodium borohydride and degraded with HCDLase. **(E)** The 2-AB labeled fraction S1; **(F)** The fraction S1 was degraded with the CSase ABC; **(G)** The 2-AB labeled S1 was degraded with the CSase ABC. **(H)** The 2-AB labeled fraction S0; **(I)** The fraction S0 was degraded with the CSase ABC, and each digest was labeled with 2-AB and analyzed by HPLC on a YMC-Pack PA-G column. The elution positions of the unsaturated disaccharides and tetrasaccharides are indicated: Δ*O*, Δ^4,5^HexUAβ1-3GalNAc; Δ*A*, Δ^4,5^HexUAβ1-3GalNAc(4S); Δ*O-O*, Δ^4,5^HexUAβ1-3GalNAcβ1-4GlcUAβ1-3GalNAc; Δ*A-O*, Δ^4,5^HexUAβ1-3GalNAc(4S)β1-4GlcUAβ1-3GalNAc; *S2*, ΔA-A-O; *S1-1*, ΔO-A-O; and *S1-2*, ΔA-O-O; *S0*, ΔO-O-O.

To elucidate the action pattern of endoVB4SF toward CS chains, the intermediates (S2 and S1) and the final product (S0) were individually collected and desalted for structural analysis through enzymatic digestion followed by HPLC analysis (Figure 5A,E,H). The results of the disaccharide composition analysis showed that S2 and S1 were composed of ΔO and ΔA units in molar ratios of 1:2 and 2:1, respectively (Figure 5B,F), while S0 contained only ΔO (Figure 5I), indicating that they were the di-, mono- and non-sulfated products derived from the digestion of ΔA-A-A by endoVB4SF. To further determine the disaccharide sequences of the intermediate S2, it was labeled with 2-aminobenzamide (2-AB) followed by digestion with the CSase ABC. Subsequently, the digest was relabeled with 2-AB and then analyzed by HPLC. Because CSase ABC cannot cleave the tetrasaccharide portion on the reducing end of hexasaccharide after 2-AB-labeling (Wang et al., 2017), the digestion of 2-AB-labeled hexasaccharide with the CSase ABC will produce a disaccharide from non-reducing end and a tetrasaccharide from reducing end. A disaccharide peak corresponding to the elution position of the disaccharide ΔA standard and a presumed tetrasaccharide peak were detected in the digests of 2-AB-labeled S2 after 2-AB relabeling, indicating that the disaccharide on the non-reducing end was the 4-*O*-sulfated A unit for S2 (Figure 5C). Based on these results, the sequences of S2 could be deduced as ΔA-(A-O/O-A). To further confirm the sequence of the tetrasaccharide on the reducing side of S2, S2 was reduced with sodium borohydride and digested with an exo-type CS/DS lyase HCDLase (Wang et al., 2017). After 2-AB labeling, the digest was analyzed by HPLC. Due to the reduction with sodium borohydride, the disaccharide released from the reducing end of S2 could not be labeled with 2-AB, and thus only the disaccharides from the tetrasaccharide portion on the non-reducing side could be labeled and detected using a fluorescence detector. The results showed that only the 2-AB-labeled ΔA unit could be detected from the digest of S2 reduced with sodium borohydride (Figure 5D), indicating that the tetrasaccharide portion on the non-reducing side of S2 had a sequence of ΔA-A. These results allowed the sequence of S2 to be deduced as ΔA-A-O. Notably, when the injected sample was reduced, the 2-AB-labeled S1 appeared as two peaks, S1-1 and S1-2, in molar ratios of approximately 2.4:1 (Figure 5E), indicating the presence of two different structures. The HPLC analysis showed that the mixture resulting from the digestion of 2-AB-labeled S1 with the CSase ABC followed by 2-AB labeling again separated into four fractions, including ΔA, ΔO-O, ΔO and ΔA-O units in a molar ratio of approximately 2.4: 2.4: 1: 1 (Figure 5G), indicating that S1 contained two structures, S1-1 and S1-2, which were ΔA-O-O and ΔO-A-O, respectively. Taken together, the digestion of hexasaccharide S3 (ΔA-A-A) showed that endoVB4SF preferentially and sequentially hydrolyzes sulfate esters from the reducing ends of CS/DS chains.

**FIGURE 6:**
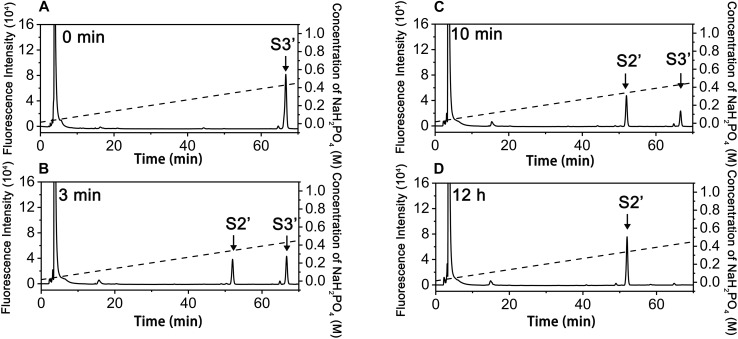
Analysis of products of hexasaccharides ΔA-A-A digested by exoFB4SF. The hexasaccharide ΔA-A-A was digested by endoVB4SF for 0 min **(A)**, 3 min **(B)**, 10 min **(C)**, and 12 h **(D)**, after which the products were labeled with 2-AB and then analyzed by anion-exchange HPLC as described as above. The elution positions of the hexasaccharide fractions are indicated: *S2’*, ΔA-A-O; and *S3’*, ΔA-A-A.

In contrast, the digestion of ΔA-A-A with exoPB4SF only yielded a single product (S2’), which eluted at the same position as S2, even after extending the treatment time to 12 h (Figure 6). The final product S2’ was pooled and sequenced using the same methods as those used for S2. The results showed that the only product, S2’, had the same structure as S2 (ΔA-A-O) (data not shown), suggesting that exoPB4SF is an exolytic sulfatase that can only hydrolyze the 4-*O*-sulfate esters of GalNAc residues located at the reducing end of CS chains.

### Crystal Structure of endoVB4SF and Structural Modeling of exoPB4SF

**FIGURE 7:**
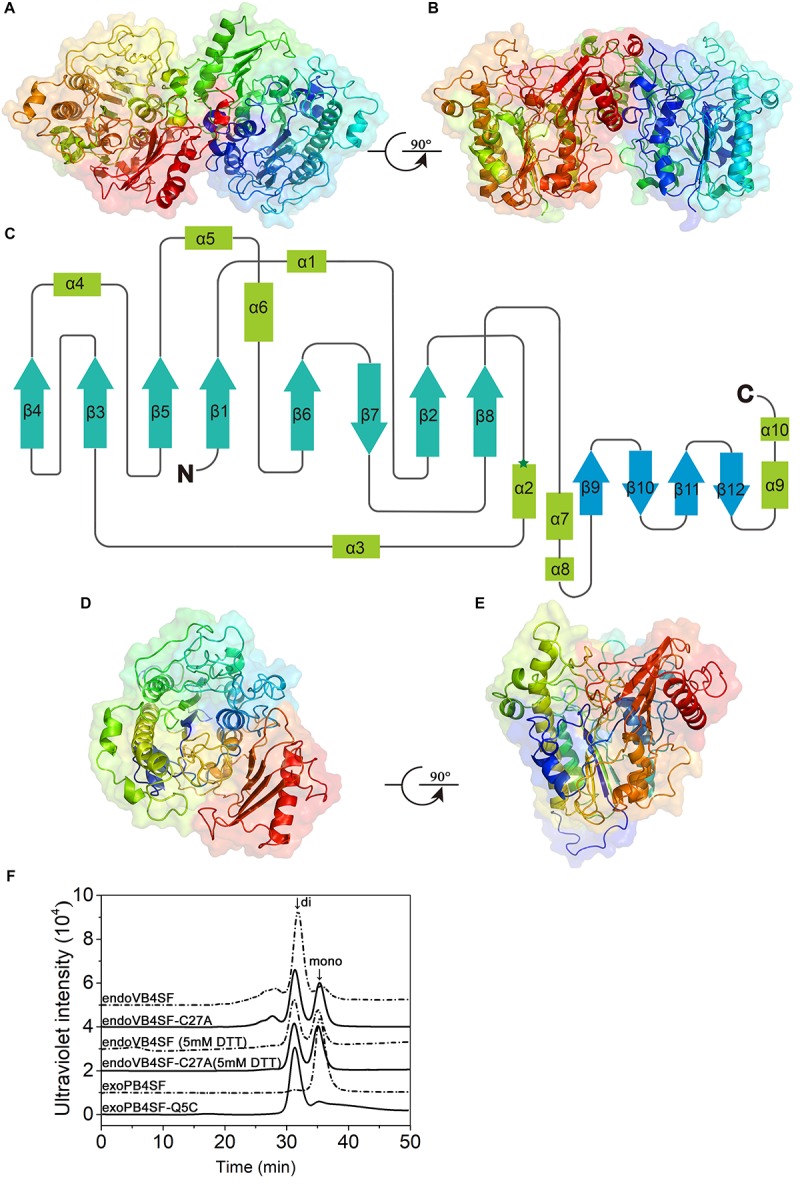
Overall structure of endoVB4SF and exoPB4SF modeling. **(A,B)** Cartoon representation of the endoVB4SF dimer in the asymmetric unit with rainbow colors. **(C)** Topological diagram of the polypeptide fold of endoVB4SF with β-sheets indicated by arrows and α-helices by rectangles. **(D,E)** Structural modeling of exoPB4SF. **(F)** Gel-filtration analysis of endoVB4SF, endoVB4SF-C27A mutant, exoPB4SF and exoPB4SF-Q5C mutant. The oligomeric states of the proteins are indicated: *di*, dimer; and *mono*, monomer.

Although the crystal structures of several CS/DS exosulfatases have been resolved, this is not the case for CS/DS endosulfatases. In this study, the crystal structure of endoVB4SF was obtained and refined at 1.95 Å resolution. EndoVB4SF adopts a globular conformation with two monomers per asymmetric unit (Figure 7A,B). The refined structure revealed that each endoVB4SF monomer folded into α/β topology containing 12 β-strands (β1–β12) and 10 α-helices (α1–α10) organized into two domains (Figure 7C). In the larger N-terminal domain (residues 20–420), the major 8-stranded β-sheets (β4, β3, β5, β1, β6, β7, β2, and β8) are sandwiched by α-helices α1, α4, α5, and α6 on one side and α-helices α2, α3, and α7 on the other side, forming the catalytic domain of the enzyme as observed in other sulfatases (Hanson et al., 2004). In the 8-stranded β-sheets, seven of them are in the parallel orientation except for strand β7, which is antiparallel to the others. In the smaller C-terminal domain (residues 421–521), four antiparallel β-strands (β9–β12) are flanked by two α-helices (α9 and α10) located on the solvent-exposed surface. The N- and C-terminal domains are closely integrated together through hydrophobic interactions to form an extensive positively charged groove, which may be important for the binding of the substrates.

Although various crystallization approaches were attempted, we failed to obtain a well-diffracted crystal for exoPB4SF. Considering that exoPB4SF shares a very high sequence identity (>80%) with endoVB4SF, its three-dimensional structure was constructed through homology modeling using I-TASSER (Figure 7D,E). The final model with the highest C score (=1.11) was selected from the 5 top models predicted. The estimated TM-score and RMSD was 0.87 ± 0.07 and 4.9 ± 3.2 Å, respectively. These parameters indicate that our structure model of exoPB4SF is of good validity.

According to the endoVB4SF structure, the two molecules in the asymmetric unit form a dimer, primarily through interactions between the C-terminal domains. The interface is approximately 1714.6 Å^2^ as predicted by PISA (Krissinel and Henrick, 2005). Size-exclusion chromatography analysis revealed that endoVB4SF primarily existed as a dimer in solution, the formation of which was significantly inhibited by the addition of DTT, indicating the existence of a disulfide bridge between the monomers at the dimer interface (Figure 7F). Compared to exoPB4SF, endoVB4SF has an extra Cys27 residue closely following the signal peptide at the N-terminal end. To investigate if this extra Cys residue is involved in the formation and stability of dimers, Cys27 was replaced by Ala (C27A) through site-directed mutagenesis. Size-exclusion chromatography analysis showed that the dimer-forming capacity of the endoVB4SF-C27A mutant was notably reduced compared to the wild-type enzyme (Figure 7F), suggesting that the Cys27 residue is involved in forming endoVB4SF dimers through the formation of intermolecular disulfide bridges between the monomers. Nevertheless, the mutation did not significantly affect the enzymatic activity toward the disaccharide ΔA or the polysaccharide CS-A (data not shown). To further investigate the role of Cys residue in the dimerization, the Gln5 of exoPB4SF, corresponding to the Cys27 in endoVB4SF, was mutated to Cys. The result showed that most of the mutant exoPB4SF-Q5C formed into dimers (Figure 7F), which confirmed the important role of Cys27 for the dimerization of endoVB4SF.

### Active Sites of endoVB4SF and exoPB4SF

**FIGURE 8:**
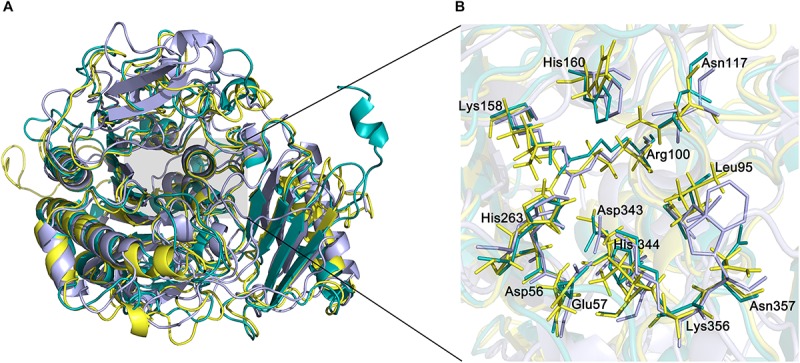
Structural alignment of endoVB4SF, ARSB and exoPB4SF. **(A)** Superposition of the endoVB4SF structure (*cyans*) to ARSB (*yellow*) and exoPB4SF (*purple*). **(B)** A more detailed view of the superposition of the active site region, colored as above. Amino acids around the active site are shown in stick mode. The labeled amino acids indicate the corresponding positions in endoVB4SF.

The active site of endoVB4SF is located in a pocket at the carboxyl end of the central parallel portion of the β sheets. The pocket is primarily formed by the amino acid residues of loops from the N-terminal domain, including Asp56, Glu57, Leu95, Cys96, Pro98, Arg100, Asn117, Lys158, His160, His263, Asp343, His344, Lys356, and Asn357. The predominance of basic residues in the active site, such as Arg100, Lys158, His160, His263, His344, and Lys356, generates a positively charged surface to facilitate the binding of negatively charged CS/DS substrates. Notably, the residue Cys96 in the N-terminal end of α2-helix is posttranslationally modified to FGly and plays a key role in the catalytic mechanism of this enzyme. Notably, all of these active-site residues are highly conserved in various sulfatases from different species. The equivalent residues involved in the catalytic center of endoVB4SF and exoPB4SF are listed in Table 4. Furthermore, a metal cation modeled as calcium was observed in the vicinity of the FGly residue and was coordinated by six atoms: four oxygen atoms from the side chains of Asp56, FGly96, and Asp343; one nitrogen from the side chain of His344; and one water molecule. A divalent metal cation, such as Ca^2+^ or Mg^2+^, has been believed to be important for the catalytic activity of sulfatases (Bruce et al., 1985; Myette et al., 2009; Rivera-Colon et al., 2012). To comprehensively analyze the tertiary structure of the exo- and endosulfatases, the structure of another exolytic 4-*O*-sulfatase ARSB (PDB code: 1fsu) (Bond et al., 1997) was downloaded and aligned with those of exoPB4SF and endoVB4SF. Through structural alignments, we observed that the orientations of the amino acid side chains at the substrate binding cavities were different in the tertiary structures of endoVB4SF, exoVB4SF and ARSB, despite the active sites of these enzymes exhibiting highly conserved secondary structures (Figure 8), indicating that the substrate-binding capacity of endo- and exo-4-*O*-sulfatases may be different.

**Table 4:** Amino acids around the catalytic center of endoVB4SF and exoPB4SF.

endoVB4SF	exoPB4SF
Phe54	Phe23
Asp56	Asp25
Leu95	Leu64
FGly96	FGly65
Pro98	Pro67
Arg100	Arg69
Asn117	Asn86
Lys158	Lys127
His160	His129
Arg179	Arg148
Trp181	Trp150
Thr201	Thr170
Asp203	Asp172
His263	His232
Ser264	Ser233
Tyr301	Tyr270
Asp343	Asp312
His344	His313
Thr355	Thr324
Lys356	Lys325
Asn357	Asn326

### Substrate Docking Simulations

**FIGURE 9:**
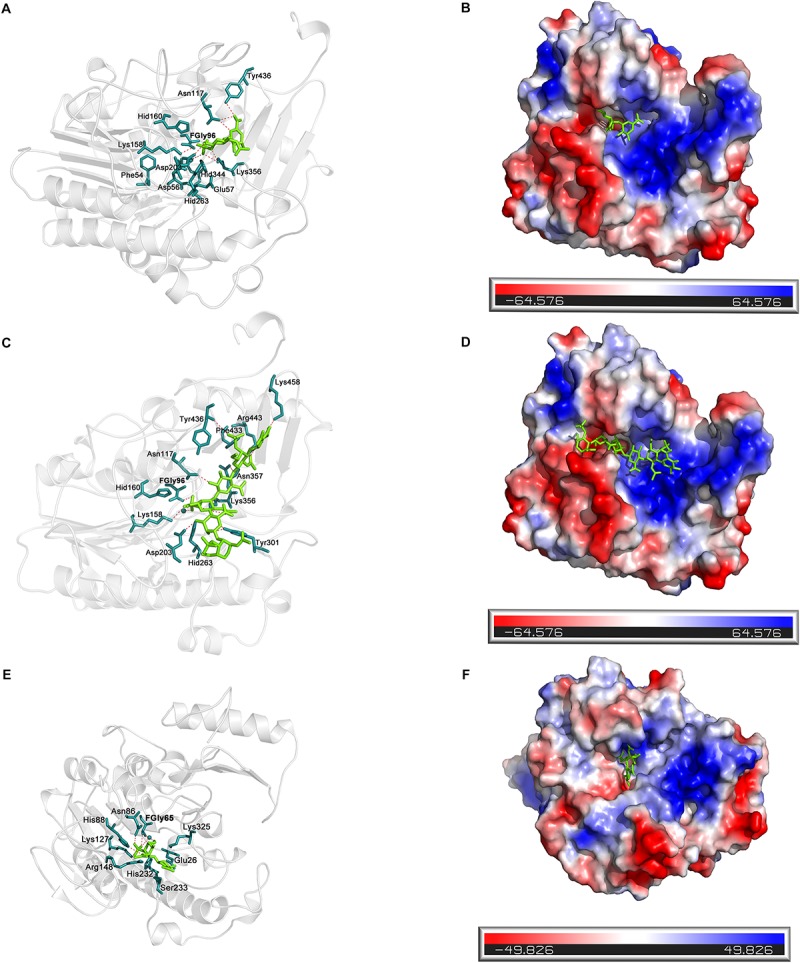
Docking results of the sugar-sulfatase complexes. **(A)** Cartoon representation of disaccharide-endoVB4SF complex. Residues constituting the binding pocket are shown as *cyans* sticks. The disaccharide is shown as *green* sticks. The metal ion is shown as *cyans* sphere. **(B)** Electrostatic surface potential of disaccharide-endoVB4SF complex. The color gradient from *red* to *blue* corresponds to the change of surface electrostatic potential. **(C)** Cartoon representation of hexasaccharide-endoVB4SF complex. Residues constituting the binding pocket are shown as *cyans* sticks. The hexasaccharide is shown as *green* sticks. The metal ion is shown as *cyans* sphere. **(D)** Electrostatic surface potential of hexasaccharide-endoVB4SF complex. The color gradient from *red* to *blue* corresponds to the change of surface electrostatic potential. **(E)** Cartoon representation of disaccharide-exoPB4SF complex. Residues constituting the binding pocket are shown as *cyans* sticks. The disaccharide is shown as *green* sticks. The metal ion is shown as *cyans* sphere. **(F)** Electrostatic surface potential of disaccharide-exoPB4SF complex. The color gradient from *red* to *blue* corresponds to the change of surface electrostatic potential.

Docking simulation studies were performed to better understand the substrate binding modes and catalytic mechanisms of endo- and exo-4-*O*-sulfatases. The unsaturated CS/DS 4-*O*-sulfated disaccharide ΔA [Δ^4,5^HexUAβ1-3GalNAc(4S)] and the hexasaccharide ΔA-A-A [Δ^4,5^HexUAβ1-3GalNAc(4S)β1-4Glc UAβ1-3GalNAc(4S)β1-4GlcUAβ1-3GalNAc(4S)] were docked into the active site of endoVB4SF and exoPB4SF, respectively. The docking results indicated that the residues Asn117, Lys158 Asp203, Lys356 and Tyr436 may participate in the binding of ΔA to endoVB4SF (Figure 9A,B). The docking scores for ΔA and ΔA-A-A to endoVB4SF were −5.24 and −7.18 kcal/mol, respectively. The lower score for ΔA-A-A suggests that endoVB4SF has a higher binding affinity toward hexasaccharide. The endo-4-*O*-sulfatase-hexasaccharide docking complex revealed that additional amino acids, including a series of positively charged residues, are involved in binding to the hexasaccharide ΔA-A-A (Figure 9C,D).

With respect to exoPB4SF, the exo-4-*O*-sulfatase-disaccharide binding mode assay revealed that the disaccharide ΔA was docked well into its active site (Figure 9E,F), with a docking score of −4.59 kcal/mol. However, the hexasaccharide ΔA-A-A could not properly dock into the active site of exoPB4SF, which may be attributable to the spatial arrangement of the amino acids in the active site. Furthermore, we observed that the substrate was fitted into the active site with the saccharide chain approximately parallel to the β-sheet core of exoPB4SF but perpendicular to the β-sheet core of endoVB4SF. These differences in substrate binding modes may lead to the different substrate length selectivity and endo-/exolytic activities observed for endoVB4SF and exoPB4SF.

## Discussion

CS/DS sulfatases are key enzymes involved in the degradation of CS/DS and are potential tools for structural and functional studies of CS/DS. However, only a few of CS/DS sulfatases have been identified from mammals and bacteria, most of which are exosulfatases, except the two endo-type sulfatases recently identified from *Bacteroides thetaiotaomicron* (Ulmer et al., 2014) and *Vibrio* sp. (Wang et al., 2015). The action pattern of CS/DS sulfatases, particular endosulfatases, has rarely been investigated. In the present study, the substrate-degrading modes of the 4-*O*- exosulfatase exoPB4SF and the 4-*O*-endosulfatase endoVB4SF, which exhibit high sequence identity, were comparatively studied through biochemical and structural analyses. The newly identified exoPB4SF from *Photobacterium* sp. shares 83% sequence identity with endoVB4SF from *Vibrio* sp. (Wang et al., 2015), but compared to endoVB4SF, exoPB4SF is a strict exolytic 4-*O*-sulfatase exhibiting a number of different features such as a wider temperature and pH adaptability and better thermostability which make it excellent tool for oligosaccharides analysis. The remarkable difference between the two highly homologous enzymes suggests that the biochemical and enzymatic features of CS/DS sulfatases are determined by both their primary and tertiary structures.

The results of previous studies showed that the *N*-acetyl- galactosamine-4-*O*-sulfatase and *N*-acetylgalactosamine-6-*O*- sulfatase from *Proteus vulgaris* could release the 4-*O*- and 6-*O*-sulfate groups from the GalNAc residues of disaccharides located at the reducing ends of various CS/DS hexasaccharides, respectively, but they had difficulty or were even unable to act upon the corresponding sulfate groups of disaccharides located in the middle and non-reducing ends of hexasaccharides (Sugahara et al., 1994; Sugahara and Kojima, 1996). The same direction selectivity was observed for endoVB4SF and exoPB4SF acting upon the CS-A-derived hexasaccharide ΔA-A-A, but endoVB4SF could easily remove the internal 4-*O*-sulfate groups of ΔA-A-A due to its endolytic activity. Interestingly, the endo-type sulfatase endoVB4SF was observed to preferentially hydrolyze the sulfate esters from the reducing to non-reducing ends of the hexasaccharide ΔA-A-A, suggesting that endosulfatases act upon CS/DS chains in a more orderly manner than endolytic lyases, which typically cleave CS/DS chains at random.

The crystal structure of endoVB4SF revealed that it adopts a globular conformation with two monomers per asymmetric unit, consistent with the results of a biophysical characterization of this enzyme (Neira et al., 2016). The results of a size exclusion chromatography analysis showed that unlike endoVB4SF, exoPB4SF existed as monomer in solution. Furthermore, the replacement of Cys27 to Ala significantly reduced the proportion of dimeric endoVB4SF in solution, suggesting that the Cys27 in endoVB4SF facilitates and stabilizes the dimer formation through the formation of a disulfide bridge, which was confirmed by the observation that the addition of DTT effectively inhibited endoVB4SF dimerization. In addition, we found that once the Gln5 of exoPB4SF, corresponding to the Cys27 in endoVB4SF, was mutated to Cys the mutant exoPB4SF-Q5C tended to form dimers. These findings suggest the important role of Cys residue in the oligomerization of some sulfatases, which is inconsistent with the results of a previous biophysical study (Neira et al., 2016). Notably, the endoVB4SF-C27A mutation could not completely inhibit dimerization, indicating that there some other interaction, such as a hydrophobic interaction, is involved in the dimerization process. Dimerization has also been observed in other sulfatases, although its biological significance is no clear (Lukatela et al., 1998; Rivera-Colon et al., 2012). We also observed that the inhibition of dimerization by site-directed mutagenesis did not influence the enzymatic activity of endoVB4SF. Thus, the exact biological function of dimer formation remains to be further elucidated.

Arylsulfatases have been reported to possess the signature motif Cys-X-Pro-X-Arg at the active site (Barbeyron et al., 2016), where the Cys residue is specifically recognized and oxidized to the aldehyde hydrate FGly [-CH(OH)_2_], catalyzed by a formylglycine-generating enzyme. Both endoVB4SF and exoPB4SF possess the Cys-X-Pro-X-Arg motif in their primary structures and, the tertiary structure revealed that the Cys96 residue in the active site of endoVB4SF was modified to the FGly residue with two geminal hydroxyl groups, indicating that CS/DS sulfatases possess the same maturation mechanism as other arylsulfatases. The FGly and other residues form the catalytic site of endoVB4SF. A bivalent metal ion was observed to be in close proximity to the hydroxyl group of FGly, which was coordinated by the residues Asp56, FGly96, Asp343, and His344. These active site residues are highly conserved in exoPB4SF and other sulfatases identified from bacteria and mammals. The highly conserved conformation of active sites suggests that endoVB4SF and exoPB4SF have the same catalytic and transesterification-elimination mechanisms as other reported sulfatases, such as ARSB (Bond et al., 1997; Waldow et al., 1999; Boltes et al., 2001). In this catalytic mechanism, the sulfate-sulfur on a saccharide residue is attacked by one of the hydroxyl groups of the FGly residue to generate a sulfated enzyme intermediate and an alcohol group, after which a sulfate group is released and the aldehyde of FGly is simultaneously regenerated through an intramolecular rearrangement. Structural alignment among endoVB4SF, exoPB4SF and ARSB shows that their catalytic cavity is rich in positively charged amino acids such as lysine, arginine and histidine, which form a positively charged catalytic region that facilitates the binding of negatively charged sugar chains. Furthermore, the catalytic cavity of endoVB4SF and exoPB4SF form a long groove while the catalytic cavity of ARSB is partially blocked by the irregular crimp from the C-terminal domain leading to a narrow substrates binding site. This difference may affect the substrate-binding and catalyzing ability of ARSB but it need to be further investigated.

Although exoPB4SF and endoVB4SF were suggested to share the same catalytic mechanism as other sulfatases, the docking results revealed that their capacities to bind substrates were different depending on the length of the CS/DS oligosaccharides. The docking models showed that small-sized substrates such as disaccharides could properly dock into the catalytic sites of both enzymes, and five highly conservative residues (FGly65, Asn86, Lys 127, His232 and Lys325 for exoPB4SF; and FGly96, Asn117, Lys158, His 263 and Lys 356 for endoVB4SF) were involved in the binding and catalysis of disaccharides (Supplementary Figures S4A,B). In contrast, large-sized substrates such as hexasaccharides could easily dock into the catalytic pocket of endoVB4SF but not exoPB4SF. The docking model of hexasaccharide-endoVB4SF revealed that additional residues were involved in the substrate binding process (Supplementary Figure S4C), where six C-terminal residues (Tyr301, Asn357, Phe433, Tyr436, Arg443, and Lys458), located on the rim of the catalytic pocket of endoVB4SF, were involved in the hexasaccharide-binding process. Interestingly, these residues are also conserved in exoPB4SF. We found that the extension direction of the side chain of these amino acids around the catalytic cavity were different. Furthermore, electrostatic surface potential diagram revealed that the catalytic center of endoVB4SF carries higher positive charges, which may facilitate the binding of negatively charged sugar chains. Therefore, we propose that the primary factor influencing the substrate selectivity of endoVB4SF and exoPB4SF is the conformation of the catalytic center in the tertiary structure. The residues at the active site of endoVB4SF adopt a more favorable conformation for the interaction of the enzyme with longer CS/DS chains than that of exoPB4SF, which may explain why endoVB4SF exhibits significant endolytic activity but exoPB4SF does not.

Overall, the action patterns of endoVB4SF and exoPB4SF were detailed characterized in this work, which will facilitate their use in structural and functional studies of CS/DS chains. The structure of endoVB4SF and molecular modeling of exoPB4SF provide structural and biochemical insight into the interaction of CS/DS chains with endo-/exo-type sulfatases, which should be instructive for gaining an understanding of the different catalytic modes between glycosaminoglycan endo- and exosulfatases.

## Data Availability

The datasets generated for this study can be found in NCBI GenBank and PDB, GenBank accession number: MK282892, PDB code:6J66.

## Author Contributions

SW conducted most of the experiments, analyzed the results, and wrote the manuscript under the guidance of FL and LG. TS performed the X-ray crystallography and structural refinement for the endoVB4SF structure. JG and JH conducted experiments for expressing the sulfatases. FL conceived the idea for the project and wrote the manuscript. All authors revised and edited the manuscript and read and approved the final manuscript.

## Conflict of Interest Statement

The authors declare that the research was conducted in the absence of any commercial or financial relationships that could be construed as a potential conflict of interest.
