# Supplementary figures and images for: Comparative Study of Two Chondroitin Sulfate/Dermatan Sulfate 4-O-Sulfatases With High Identity
Source: Front Microbiol. 2019 Jun 12;10:1309. doi: 10.3389/fmicb.2019.01309 (PMC6581707; doi:10.3389/fmicb.2019.01309)

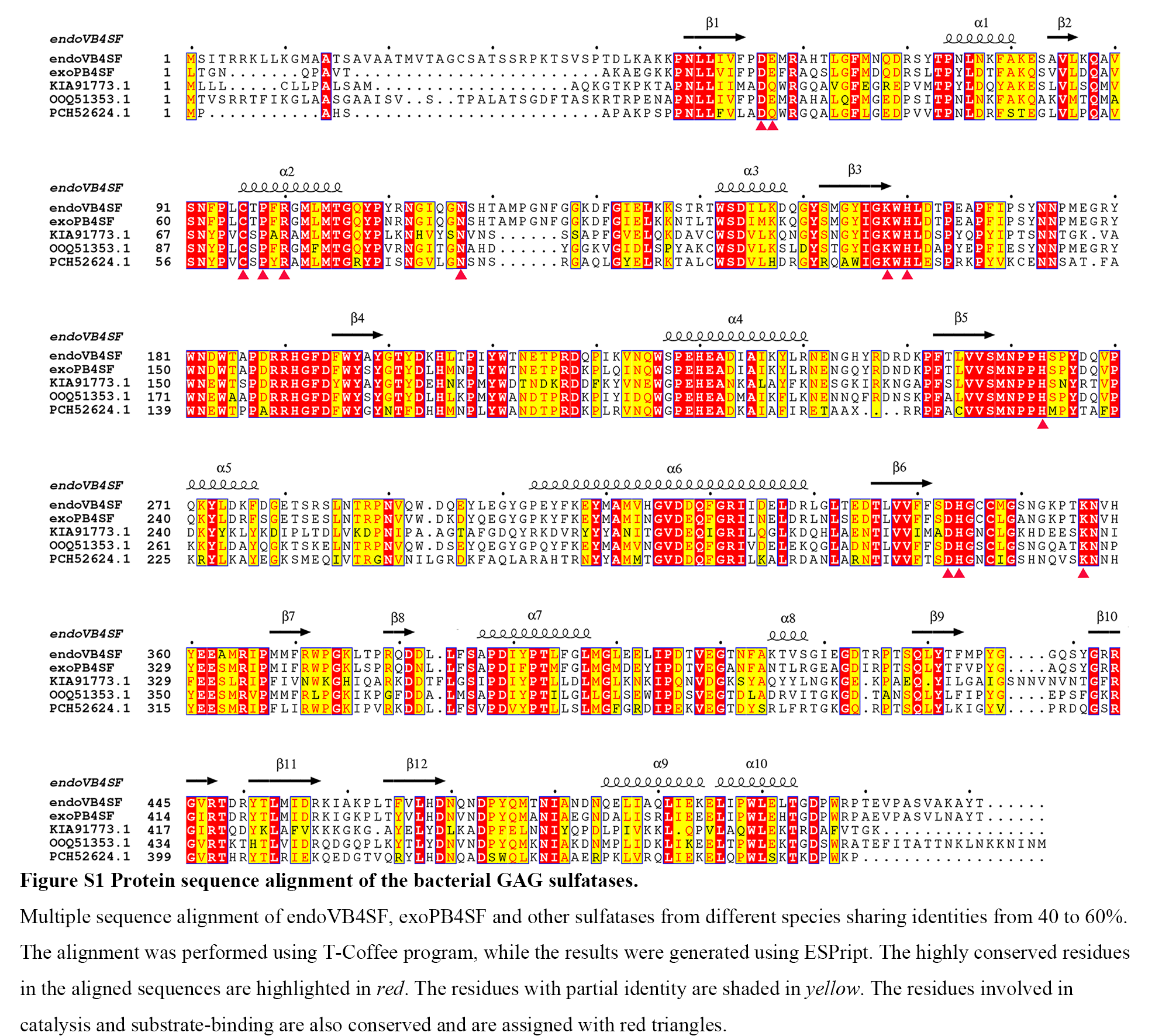

Supplement: Supplementary file 1 [file Image_1.TIF]

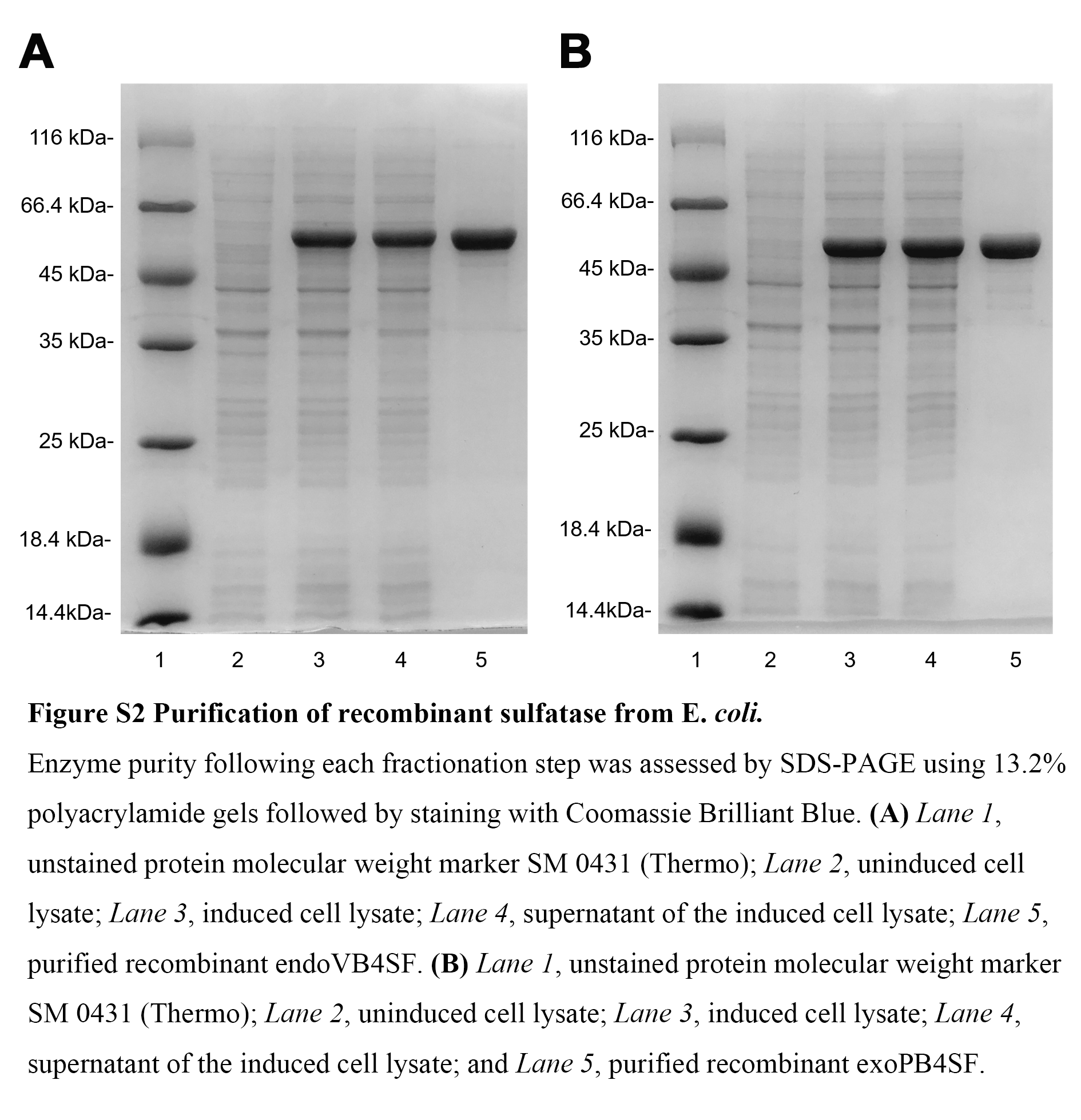

Supplement: Supplementary file 2 [file Image_2.TIF]

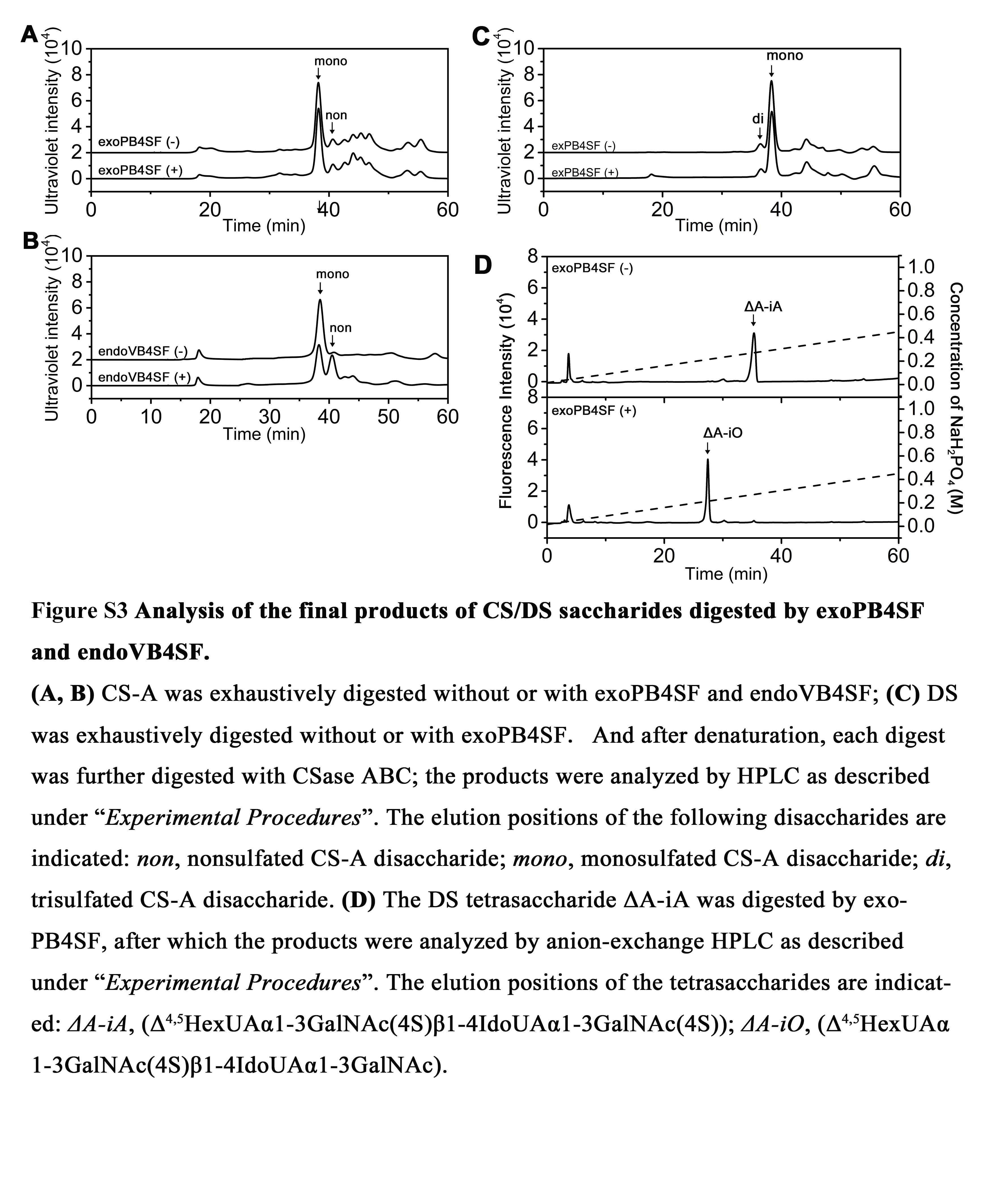

Supplement: Supplementary file 3 [file Image_3.TIF]

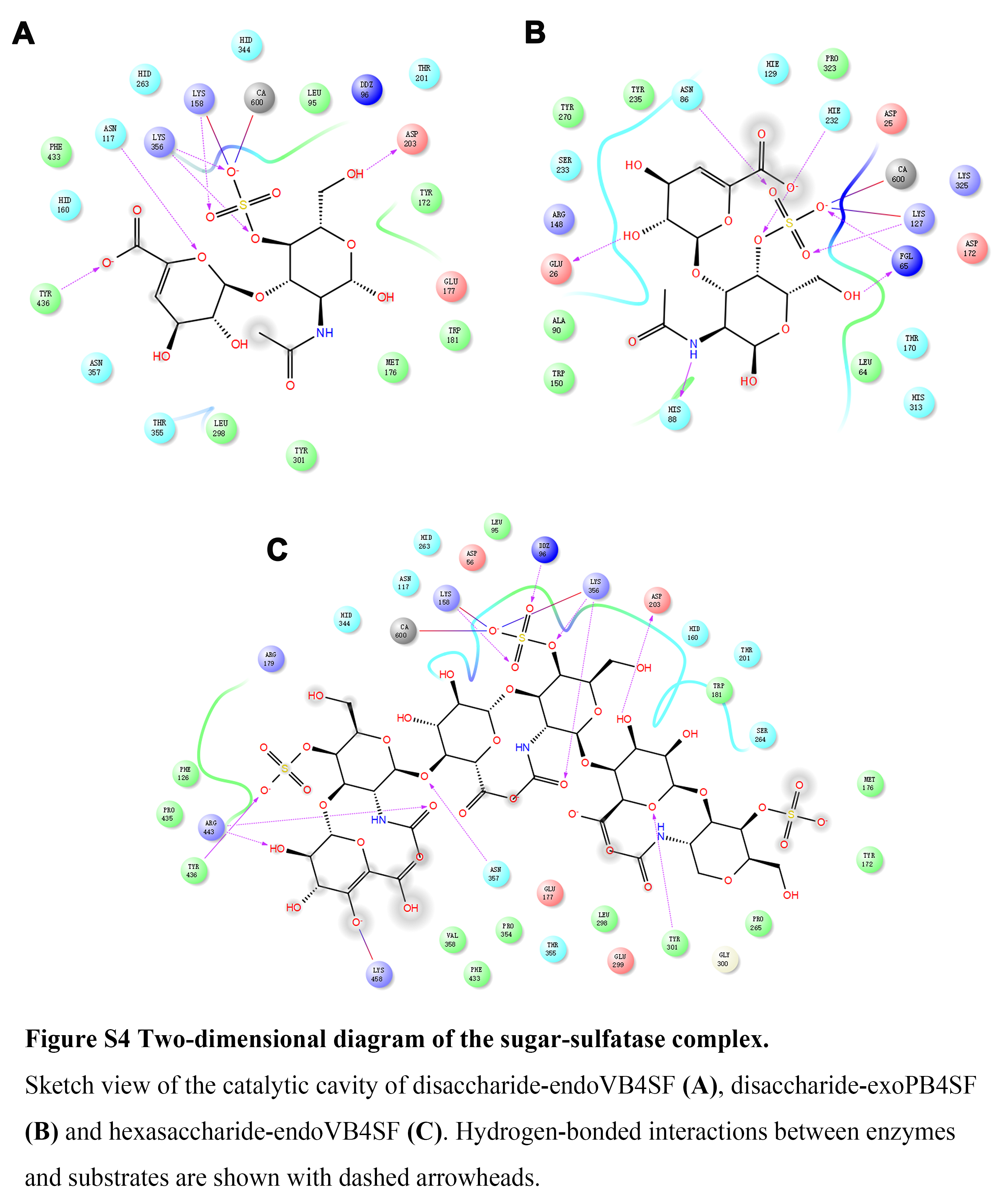

Supplement: Supplementary file 4 [file Image_4.TIF]
